# MMP-2 mediates local degradation and remodeling of collagen by annulus fibrosus cells of the intervertebral disc

**DOI:** 10.1186/ar4224

**Published:** 2013-04-27

**Authors:** Anshu Rastogi, Hyunchul Kim, Julianne D Twomey, Adam H Hsieh

**Affiliations:** 1 Fischell Department of Bioengineering; University of Maryland, College Park, Jeong H. Kim Engineering Building, Rm 2330, College Park, MD 20742, USA; 2 Department of Orthopaedics, School of Medicine; University of Maryland, Baltimore, 22 South Greene Street; Baltimore, MD 21201, USA

**Keywords:** Intervertebral disc, Matrix metalloproteinase, Collagen, Remodeling, RNA interference

## Abstract

**Introduction:**

Degeneration of the intervertebral disc (IVD) is characterized by marked degradation and restructuring of the annulus fibrosus (AF). Although several matrix metalloproteinases (MMPs) have been found to be more prevalent in degenerate discs, their coordination and function within the context of the disease process are still not well understood. In this study, we sought to determine whether MMP-2 is associated with degenerative changes in the AF and to identify the manner by which AF cells use MMP-2.

**Methods:**

Two established animal models of disc degeneration, static compression and transannular needle puncture of rodent caudal discs, were examined for MMP-2 immunopositivity. With lentiviral transduction of an shRNA expression cassette, we screened and identified an effective shRNA sequence for generating stable RNA interference to silence MMP-2 expression in primary rat AF cells. Gelatin films were used to compare gelatinase activity and spatial patterns of degradation between transduced cells, and both noninfected and nonsense shRNA controls. The functional significance of MMP-2 was determined by assessing the ability for cells to remodel collagen gels.

**Results:**

Both static compression and 18-g annular puncture of rodent caudal discs stimulated an increase in MMP-2 activity with concurrent lamellar disorganization in the AF, whereas 22-g and 26-g needle injuries did not. To investigate the functional role of MMP-2, we established lentivirus-mediated RNAi to induce stable knockdown of transcript levels by as much as 88%, and protein levels by as much as 95% over a 10-day period. Culturing transduced cells on gelatin films confirmed that MMP-2 is the primary functional gelatinase in AF cells, and that MMP-2 is used locally in regions immediately around AF cells. In collagen gels, transduced cells demonstrated an inability to remodel collagen matrices.

**Conclusions:**

Our study indicates that increases in MMP-2 observed in human degenerate discs are mirrored in experimentally induced degenerative changes in rodent animal models. AF cells appear to use MMP-2 in a very directed fashion for local matrix degradation and collagen remodeling. This suggests that MMP-2 may have a functionally significant role in the etiology of degenerative disc disease and could be a potential therapeutic target.

## Introduction

Various physiologic and pathophysiologic processes of connective tissues involve cell-mediated matrix-remodeling events, including tissue growth, repair, and degeneration. During remodeling, extracellular matrix (ECM) turnover rates are increased [1], causing shifts in tissue architecture and composition. Although a number of molecules are thought to regulate this process, matrix metalloproteinases (MMPs) are believed to be particularly important in ECM degradation [2-4]. In addition to being the key mediators of tissue remodeling, MMPs are also known to be involved in cell proliferation, migration, differentiation, angiogenesis, apoptosis, and host defense [5].

A notable example of chronic pathophysiologic remodeling occurs in the intervertebral disc (IVD) of the spine during degenerative disc disease (DDD). In the annulus fibrosus (AF), there is an acceleration of age-related matrix changes, which are thought to be caused by enhancement of catabolic processing of the ECM [3,4,6,7]. Several MMPs have been shown to be expressed at elevated levels in the AF of diseased discs [2]. One of these is MMP-2, a gelatinase that participates in the secondary breakdown of collagen during remodeling [2,4,8,9]. Activation of MMP-2 above endogenous baseline levels has been found to be induced by mechanical stress [10,11]. However, the functional roles of MMP-2 in disc health and degeneration remain unclear. An improved understanding of the molecular mechanisms involved in DDD would contribute to the development of interventional strategies [7] to mitigate degenerative processes.

In recent years, RNA interference (RNAi) has become a powerful and accessible tool for manipulating cellular function, particularly with the use of small RNAs for sequence-specific gene silencing [12,13]. Although extensive studies are required to elucidate unanticipated effects, the capability of RNAi to induce both transient and stable silencing [12,14] contribute to its promise as a technology for treating human disease.

We had previously observed that mechanical overloading of murine IVDs results in altered MMP-2 activation patterns [10,15]. As a first step toward improving our understanding of MMPs in DDD, this study demonstrates that, in a rat annular-injury model of degenerative changes, discs similarly exhibit an upregulation of MMP-2. To investigate the functional implications of MMP-2, we then validated an approach to silence MMP-2 gene expression by using lentivirus-mediated RNAi in rat AF primary cell cultures. We found that MMP-2 silencing inhibited degradation of gelatin immediately surrounding cells and impaired the ability of cells to remodel collagen gels. To our knowledge, this is the first study to use small-hairpin RNAs (shRNAs) to achieve stable knockdown of MMP-2. Results suggest that MMP-2 is used locally by cells to drive changes in ECM structure and function.

## Materials and methods

### Surgical procedure

Sprague-Dawley rats (6 to 9 months old; Taconic Farms, Germantown, NY, USA) were subjected to annular puncture to induce degenerative changes [16], after approval from the Institutional Animal Care and Use Committee at the University of Maryland, College Park. Rats were anesthetized by using isoflurane, and the c6-7 motion segment was identified, marked, and confirmed by using fluoroscopy. A subcutaneous injection of analgesic (buprenorphine, 0.03 mg/0.1 ml) and an intramuscular injection of antibiotic (fluoroquinolone, 2.5 mg/0.1 ml) were given to the rat. The diameter of the tail at the puncture site was measured with calipers to determine the necessary depth of needle insertion. After establishing a sterile field and scrubbing with povidone/iodine (Betadine)-isopropyl alcohol, a cranial-caudal skin incision was made on the dorsal aspect of the tail in the proximity of c6-7. Soft tissues were parted to identify the c6-7 disc, and a sterile tapered hypodermic needle (26-g, 22-g, or 18-g) was marked to the depth corresponding to the radius of the tissue, and then carefully inserted by hand. Fluoroscopy was used to confirm proper position and depth. For sham surgeries, the skin incision was made, but discs were left uninjured. Incisions were then sutured closed. After surgery, rats were revived and returned to normal cage activity for 2 weeks, with daily observation for pain and distress. Rats were initially given an injection of analgesic (buprenorphine, 0.03 mg/0.1 ml), after which analgesic (buprenorphine at 0.05 mg/kg rat), antiinflammatory (Carprofen at 5 mg/kg rat), and antibiotic (cephalexin at 60 mg/kg rat) were administered, if necessary, in accordance with our pain/distress assessments and consultation with the veterinarian.

### Immunohistochemistry

After 2 weeks, the rats were killed by carbon dioxide asphyxiation (6 to 8 L/min until loss of consciousness, followed by 12 L/min for 10 minutes) and bilateral thoracotomy. Motion segments were then dissected from tails, and then fixed in formalin, decalcified in a solution containing 10% (wt/vol) sodium citrate and 20% formic acid, and processed in graded ethanol and xylene baths before paraffin embedding (TP1020/EG1160; Leica Microsystems, Buffalo Grove, IL, USA). Paraffin blocks were sectioned at 6 μm by using a microtome (HM355; Microm/Thermo Fisher Scientific, Waltham, MA, USA). Slides containing serial sections close to the midsagittal regions of punctured or control discs were deparaffinized and rehydrated. Sections were blocked with goat serum for 1 hour, washed, and then incubated with primary antibody against MMP-2 (clone SPM346; Abcam, Cambridge, MA, USA) at either 0 (no Ab control), 1, or 10 μg/ml. A biotin-labeled goat anti-mouse IgG secondary antibody was used with horseradish peroxidase-conjugated streptavidin (Vectastain ABC; Vector Laboratories, Burlingame, CA, USA), and 3,3 -diaminobenzidine substrate to develop a color reaction. Each batch of 26-g, 22-g, 18-g, and nonpunctured discs was immunostained concurrently under identical conditions, and images were captured under identical exposure conditions to ensure valid qualitative comparisons of staining intensity.

### Primary cell isolation and culture

Healthy AF tissues were freshly harvested under sterile conditions from Sprague-Dawley rat caudal discs immediately after euthanasia, performed as described earlier according to procedures approved by the Institutional Animal Care and Use Committee at the University of Maryland, College Park. Tissues were minced and digested overnight in culture media containing 3 mg/ml collagenase. Digests were then centrifuged, and cells were resuspended in fresh culture media and plated in tissue-culture flasks. Cells were cultured until passage 3 in DMEM containing 10% FBS, 100 U/ml penicillin, and 100 µg/ml streptomycin at 37°C with 5% CO_2_, with media changes 3 times per week.

In preliminary experiments, we found that rat AF cells express similar levels of types I and II collagen, sox9, and aggrecan during low-passage culture. Passage 3 cells were selected as a balance between adequate cell expansion and retention of cell phenotype.

### Lentivirus construct preparation

Five 21- to 23-nt long sequences (Table 1) were identified from the rat *MMP2 *mRNA [GenBank: NM_031054] for designing candidate shRNA constructs (designated shMMP2a-e). Stem-loop-stem sequences corresponding to each shRNA construct were cloned into pENTR/U6 (Invitrogen, Carlsbad, CA, USA), which drives production of shRNA through a human U6 expression cassette. Plasmids were then genetically recombined with pLenti6/BLOCK-iT-DEST (Invitrogen), which confers blasticidin resistance. A nonsense control (shNon), containing a scrambled sequence, was also cloned. Replication-deficient lentivirus was then produced by packaging the vector in 293FT embryonic kidney cells by using Lipofectamine 2000 and manufacturer-supplied packaging mix (Invitrogen). Virus-containing supernatants were harvested 72 hours later, sterile filtered, and stored at -80°C. Experiments used either a mix of transduced and nontransduced cells, or a blasticidin-purified population of transduced cells.

**Table 1:** Matrix metalloproteinase 2 (MMP-2) shRNA sequences

Sequence name	Top and bottom "stem-loop-stem" sequences, 5 to 3
shMMP2a	CACCGCTGAAGGACACCCTCAAGAACGAATTCTTGAGGGTGTCCTTCAGC
	
	AAAAGCTGAAGGACACCCTCAAGAATTCGTTCTTGAGGGTGTCCTTCAGC

shMMP2b	CACCGCCGGGATAAGAAGTATGGATTCTCGAAAGAATCCATACTTCTTATCCCGG
	
	AAAACCGGGATAAGAAGTATGGATTCTTTCGAGAATCCATACTTCTTATCCCGGC

shMMP2c	CACCGCTGTGTTCTTCGCAGGGAATCGAAATTCCCTGCGAAGAACACAGC
	
	AAAAGCTGTGTTCTTCGCAGGGAATTTCGATTCCCTGCGAAGAACACAGC

shMMP2d	CACCGCAATACCTGAACACTTTCTACGAATAGAAAGTGTTCAGGTATTGC
	
	AAAAGCAATACCTGAACACTTTCTATTCGTAGAAAGTGTTCAGGTATTGC

shMMP2e	CACCGTGGTGGTCACAGCTATTTCTTCCGAAGAAGAAATAGCTGTGACCACCA
	
	AAAATGGTGGTCACAGCTATTTCTTCTTCGGAAGAAATAGCTGTGACCACCAC

shNon	CACCGCCGATTAGCTGATCGTGCTTAGTCGAAACTAAGCACGATCAGCTAATCGG
	
	AAAACCGATTAGCTGATCGTGCTTAGTTTCGACTAAGCACGATCAGCTAATCGGC

### Cell transduction

AF cells were plated in six-well tissue-culture plates in complete media (DMEM, supplemented with 10% FBS and 1% penicillin-streptomycin; Gibco/Invitrogen, Carlsbad, CA, USA) at 50% confluence (150,000 cells/well). Each of the six viral constructs (shMMP2a-e, shNon) was added to one well each, at multiplicity of infection (MOI) of 0.1, along with 6 µg/ml Polybrene (Sigma, St. Louis, MO, USA) to facilitate transduction. Viral particles were removed the next day through aspiration of the culture medium, and replaced with complete culture medium. Blasticidin (Invitrogen) was added in appropriate samples to select for transduced cells the day after virus removal, at a concentration of 8 μg/ml, based on preliminary experiments of AF cell sensitivity to blasticidin. Media was changed every 3 days. In appropriate samples, each media change contained blasticidin. Each time point also included a corresponding noninfected control (plated cells not exposed to viral particles).

Three separate experiments were conducted to determine *MMP2 *expression for each of the five shMMP viral constructs. Experiment 1 was conducted to determine the mixed-population response. As such, cells were infected and harvested at 1, 4, 7, and 10 days after virus removal without selection. Experiment 2 examined the response of the pure transduced cell population. After infection, cells were harvested at 1, 4, 7, and 10 days under continuous blasticidin treatment. Experiment 3 assessed the stability of gene silencing by subjecting cells to a 10-day continuous blasticidin treatment, followed by culture in blasticidin-free complete media for an additional 1, 4, 7, and 10 days. At each time point, samples were collected in Tris-HCl, pH 7.5 to 8.0 by using a cell scraper. Each harvested sample was divided into three groups to measure (a) DNA content, (b) MMP-2 content, and (c) gene expression. Conditioned media was also harvested from samples for measurement of MMP-2 content. All samples were maintained at -80°C until use.

### *MMP2 *expression

MMP-2 content was quantified by using a microplate-based activity assay (R&D Systems Inc., Minneapolis, MN, USA). One fourth (25%) of each harvested sample (see Cell transduction) was thawed, pulverized with a pestle, and then loaded into microplates along with conditioned media samples and standards. Protein levels of MMP-2 were obtained according to the manufacturer's protocol. Another one fourth (25%) of each harvested sample was subsequently thawed, subjected to five additional freeze/thaw cycles for cell lysis, and loaded into a microtiter plate along with standards generated from Lambda DNA. PicoGreen (Invitrogen) reagent was then added to all wells to measure DNA content. Absorbance (MMP-2) and fluorescence (DNA) measurements were made with a SpectraMax M5 plate reader (Molecular Devices, Sunnyvale, CA, USA). All measured MMP-2 protein levels were normalized to DNA content from the corresponding sample, determined by using PicoGreen, to examine comparable protein knockdown for each population.

For quantitative RT-PCR, the remaining 50% of each thawed sample was used for RNA isolation (RNeasy Micro; Qiagen, Inc., Valencia, CA, USA). Total RNA was then reverse transcribed (Ambion/Applied Biosystems, Austin, TX, USA), and the resulting cDNA was used for SYBR Green-based real-time PCR (MyiQ; Bio-Rad Laboratories, Hercules, CA, USA) to quantify expression of GAPDH and MMP-2. Primers for each were designed for rat genes (Table 2) by using Primer3 software [17]. Results were analyzed by using the ΔΔCt method [18]. These ΔΔCt values were then expressed as relative changes in mRNA levels (fold difference) through the exponential relation 2^-ΔΔCt^. All gene-expression data were statistically analyzed (SPSS 14.0, Chicago, IL, USA) by independent-sample *t *tests (critical significance level, α = 0.05), comparing the experimental condition with wild-type controls to determine whether treatments had an effect on MMP-2 gene expression.

**Table 2:** Sequences of primers used for RT-PCR analysis

Gene name	Forward and reverse primers	**GenBank accession no**.	Length of product (bp)
*GAPDH*	5 -AACCCATCACCATCTTCCAG-3	NM_017008	197
	5 -GTGGTTCACACCCATCACAA-3		

*MMP2*	5 -AGCTCCCGGAAAAGATTGAT-3	NM_031054	180
	5 -TCCAGTTAAAGGCAGCGTCT-3		

### Gelatin films

A 5% (wt/vol) gelatin solution was pipetted onto glass slides and allowed to air dry. Once cooled, the slides were chemically crosslinked in 4% formalin for 1 hour at room temperature, washed extensively with sterile PBS, and then stored overnight in PBS at 4°C. The next day, the PBS was removed and slides were equilibrated in complete cell culture media for 30 minutes before cell seeding.

Separate populations of AF cells infected with the most effective of the five shRNA constructs against *MMP2 *(hereafter designated shMMP2), and the nonsense shRNA construct (shNon) were treated with blasticidin to obtain pure populations of transduced cells. Transduced cells (*n *= 4 for shMMP2 and shNon each) and noninfected control cells (*n *= 4) were seeded onto the films at densities of approximately 200,000 cells/film. The cell suspension was allowed to settle for 30 minutes before cell-culture media was added. Gelatin films without cells served as negative controls (*n *= 4). After 4 days in normal culture conditions, media was removed from the dish, and films were rinsed once with PBS, stained with 1% Ponceau S for 1 minute, and washed with DI water for 5 minutes on a shaker. Each replicate of gelatin films was stained in parallel and imaged immediately after staining under identical exposures to standardize comparisons. Extent of degradation of gelatin films was quantified by using Image J (National Institutes of Health, Bethesda, MD, USA). In brief, images were first segmented by thresholding to distinguish degraded (white) from nondegraded (red) regions. The areas of these regions were then used to calculate a percentage degradation equal to the ratio of degraded area to total area in each field of view. Statistical analysis (SPSS) was performed by using a Kruskal-Wallis test with Games-Howell *post hoc *analysis.

### Collagen gels

Three-dimensional type I collagen gels were made from rat-tail collagen (BD Biosciences, San Jose, CA, USA) at a concentration of 3 mg/ml by following the manufacturer's protocols. All components were prechilled and sterile. In brief, the collagen I stock solution was diluted in 10× PBS (Gibco/Invitrogen), sterile water, and 1N NaOH to achieve a final concentration of 3 mg/ml collagen in 1× PBS at pH 7.4. The collagen solution was kept on ice until use.

AF cells were infected with shMMP2 or shNon virus, as described previously. Two separate populations of cells were used for each of the two viruses, one of which was treated with blasticidin to obtain a pure population of only transduced cells, whereas the other was used as a mixed population of transduced and noninfected cells (*n *= 3 for each). Noninfected cells served as positive control (*n *= 3), with collagen gels without cells (*n *= 3) serving as negative control. Cells were trypsinized, pelleted, and resuspended in collagen solution at 500,000 cells/ml. The collagen-cell solution was pipetted into custom-made dog-bone-shaped molds (3 mm thick), fabricated to be 15 mm in width at the grips with a 5-mm-wide × 10-mm-long gauge region, placed in petri dishes. Polypropylene mesh (Small Parts, Inc., Logansport, IN, USA) with approximately 300-μm pore size was embedded in the collagen at each end of the mold for gripping. Collagen gel constructs were incubated for 90 minutes at 37°C to allow polymerization, and then placed in six-well plates in free-floating culture with complete media. Gels were cultured for 7 days, at which point, they were used either for mechanical testing to ascertain changes in material properties, or fixed for histology to evaluate structural changes.

### Mechanical testing and histology

**Figure 1:**
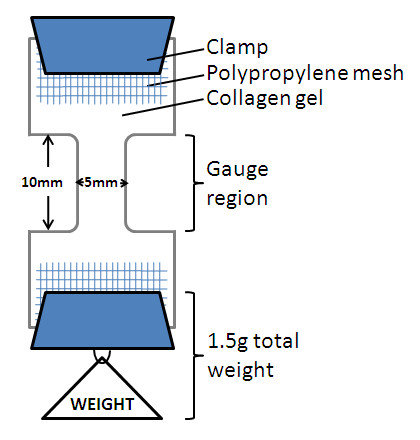
**Schematic representation of collagen gel mechanical testing**. Collagen gels were clamped on each end at the mesh, and creep testing was performed in tension by using hanging weights. An initial weight of 1.5 g was added, followed by increments of 0.5 g at 2-minute intervals until failure. The cross-sectional area, measured before loading, was used to calculate applied engineering stress, and analysis of deformations in individually captured frames was used to calculate the engineering strain. Mechanical viscoelastic behavior was quantified by fitting data to the standard linear viscoelastic solid-model equation for creep loading.

For mechanical testing, gels were removed from culture, and their widths and thicknesses at the gauge region were measured by using calipers. Gels were clamped on each end at the mesh, and creep testing was performed in tension by using hanging weights (Figure 1). An initial weight of 1.5 g was added, followed by increments of 0.5 g at 2-minute intervals until failure. For each specimen, applied engineering stress was calculated by using cross-sectional areas measured before loading. Deformations were captured by using a video system, and engineering strain computed in the gauge region by manual analysis of individual image frames. Mechanical viscoelastic behavior was quantified by fitting data to the standard linear viscoelastic solid-model equation for creep loading (Eq 1) by using MATLAB (Mathworks, Natick, MA, USA) to obtain values for parameters E_1 _and E_2 _(measures of elasticity), and μ (measure of viscosity).

(1) $$\varepsilon \left(t\right)=\sigma_{0}\left[\frac{1}{E_{2}}+\left(\frac{1}{E_{1}+E_{2}}-\frac{1}{E_{2}}\right)e^{-\frac{E_{1}E_{2}}{\mu E_{1}+\mu E_{2}}t}\right]$$

The short-term effective modulus (that is, as t → 0), E_1 _+ E_2_; the long-term effective modulus (that is, as t →∞), E_2_; the viscous parameter, μ; and the stress at collagen-gel rupture were used for comparison among experimental groups. One-way analysis of variance with Tukey *post hoc *tests was performed (SPSS) to determine whether differences among groups were statistically significant (α = 0.05).

For histology, gels were fixed overnight in 10% buffered formalin. Samples were then dehydrated through sequential ethanol baths, cleared in xylene, and infiltrated with paraffin. Paraffin blocks were cut to obtain 6-μm sections, which were stained with Safranin-O/Fast green to evaluate changes in collagen structure.

## Results

### Disruption of mechanical integrity of the AF enhances MMP-2 expression

**Figure 2:**
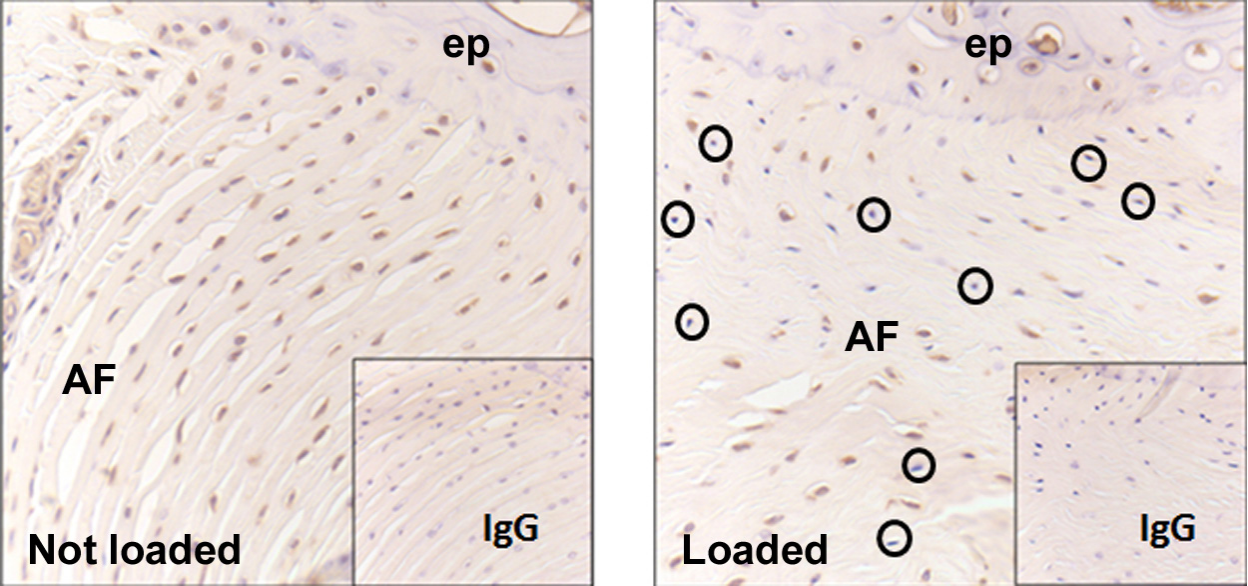
**Static compression induces matrix metalloproteinase 2 (MMP-2) activation**. Immunohistochemical staining using an antibody (CA719E3C; Labvision, Fremont, CA, USA) that recognizes only the latent MMP-2 zymogen in nonloaded (left) and loaded (right) IVDs. These data are unpublished, but presented in a poster [15]. The loss of positive staining from cells of loaded discs coincided with previously observed increases in activation of MMP-2, as determined with enzyme-linked immunosorbent assay (ELISA) [10].

**Figure 3:**
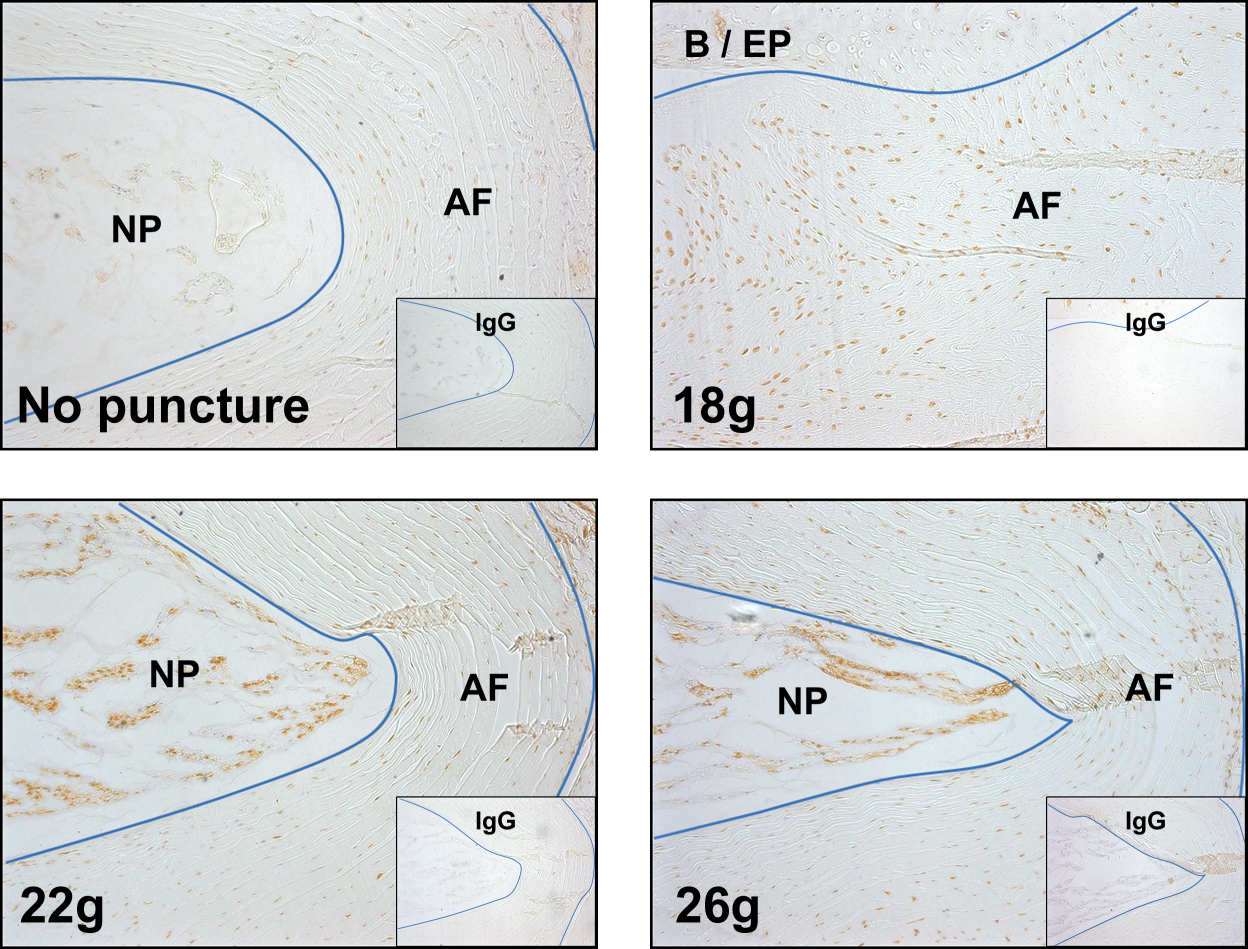
**Transannular puncture injury upregulates matrix metalloproteinase 2 (MMP-2)**. Immunohistochemical staining of MMP-2 in rat caudal discs subjected to transannular punctures by using hypodermic needles (18-g, 22-g, and 26-g) or subjected to sham surgery (no puncture). Although AF cells in all tissue sections exhibited some immunopositivity, more positive cells were found, and the qualitative intensity of staining was markedly higher in punctured discs, particularly for 18-g needles. We previously showed that 18-g hypodermic needle punctures are associated with a greater incidence of degenerative changes in the AF [16].

We previously presented data showing localized MMP-2 activation when murine caudal discs are subjected to compressive loads that induce degenerative changes in the AF [15] (images reproduced in Figure 2). After our recent findings of greater AF disorganization caused by needle-puncture injury-induced IVD depressurization [16], we postulated that MMP-2 might be involved in these observed degenerative changes as well. At 2 weeks, we detected strong immunopositivity for MMP-2 in rat caudal IVDs punctured with 18-g hypodermic needles, and distinctly weaker staining in 22-g and 26-g punctured discs, which were similar to nonpunctured controls (Figure 3). Based on these results, we sought to investigate the functional role of MMP-2 in cell-mediated collagen remodeling by AF cells.

### *MMP2 *expression can be silenced in primary AF cells through stable RNA interference

To elucidate the role of MMP-2 in matrix organization, we set out to develop an experimental system for silencing MMP-2 in rat IVD cells to complement our *in vivo *studies. After constructing five lentiviral vectors for expressing shRNA to target *MMP2*, designated shMMP2a-e, and a nonsense control shNon (Table 1), we performed a preliminary screen to identify an effective silencing construct (data not shown). We examined the knockdown of *MMP2 *expression in (a) mixed (transduced and nontransduced) populations, (b) pure (blasticidin-selected) populations, and (c) long-term cultures of pure populations. All constructs, except shMMP2a, led to decreased mRNA and protein levels, as determined with qRT-PCR and MMP-2 activity assay, respectively. In Experiment 1, the mixture of transduced and nontransduced cells exhibited downregulation, peaking at 4 days after infection, but then progressively climbed higher through 10 days, presumably because of proliferation of nontransduced cells. In Experiments 2 and 3, downregulation remained more consistent over time. Overall, shMMP2c and d were more successful in knocking down gene expression compared with the other constructs, with up to 80% silencing observed for both. Protein levels for shMMP2b-e were all markedly lower than shNon controls. Based on the composite data, we selected shMMP2d (hereafter designated shMMP2) for use in subsequent experiments.

**Figure 4:**
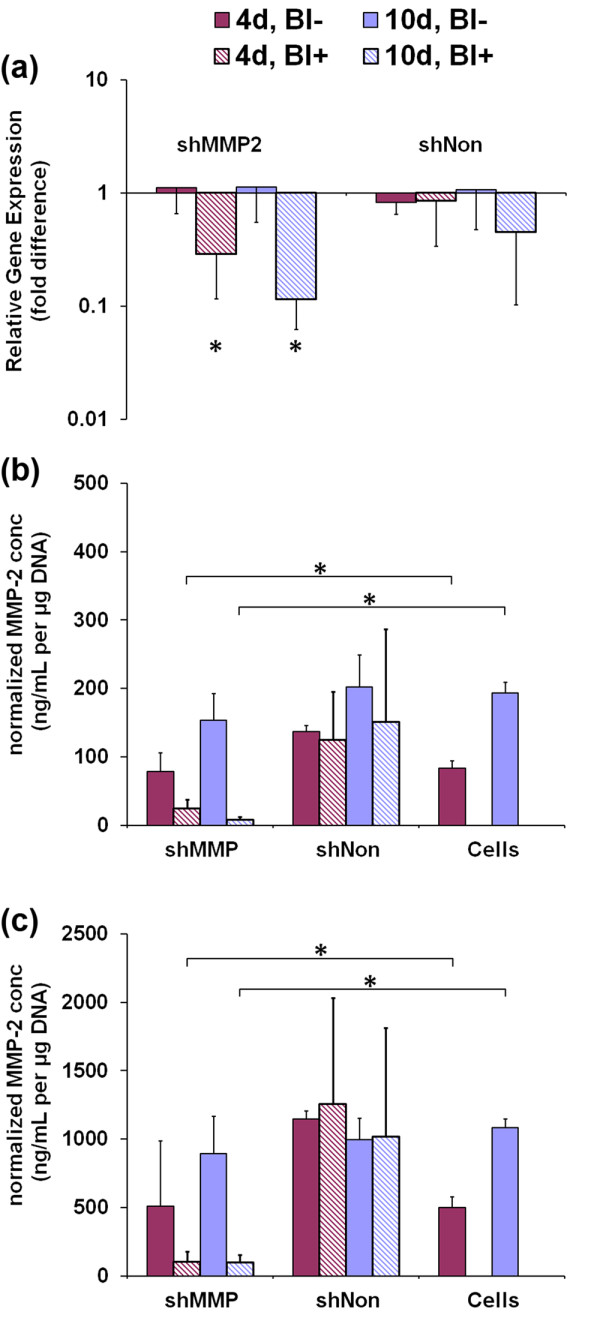
**Stable silencing of matrix metalloproteinase 2 (MMP-2) by using shRNA-mediated RNAi**. Silencing of *MMP2 *in primary rat caudal annulus fibrosus (AF) cells at 4 and 10 days after lentivirus infection, with and without treatment with blasticidin to select for pure populations of transduced cells. **(a) **Relative expression of treatment groups compared with noninfected controls, as quantified with real-time RT-PCR. At 4 and 10 days, *MMP2 *transcript levels were significantly decreased by 70% and 88%, respectively, in blasticidin-treated cultures (*P < 0.03). **(b) **Total protein levels for MMP-2 in cell lysates and **(c) **in conditioned media. At 10 days, total MMP-2 is 95% and 90% lower (*P < 0.05) in blasticidin-treated cell lysates and culture media, respectively, compared with that in noninfected controls (Cells).

Replicates using three additional independent primary cell isolations (*n *= 3) were then performed by using shMMP2 and shNon to validate results of the screening experiments. As before, compared with time-matched wild-type cells, blasticidin-selected cells transduced with shMMP2 contained significantly lower mRNA (*P *< 0.03; Figure 4a), as well as significantly lower cell-associated (*P *< 0.02; Figure 4b) and secreted levels of MMP-2 (*P *< 0.05; Figure 4c). At each time point, all mixed populations of shMMP2-infected cells and shNon-infected cells were not different from corresponding wild-type controls.

### RNAi of *MMP2 *expression inhibits local matrix degradation in AF cells

**Figure 5:**
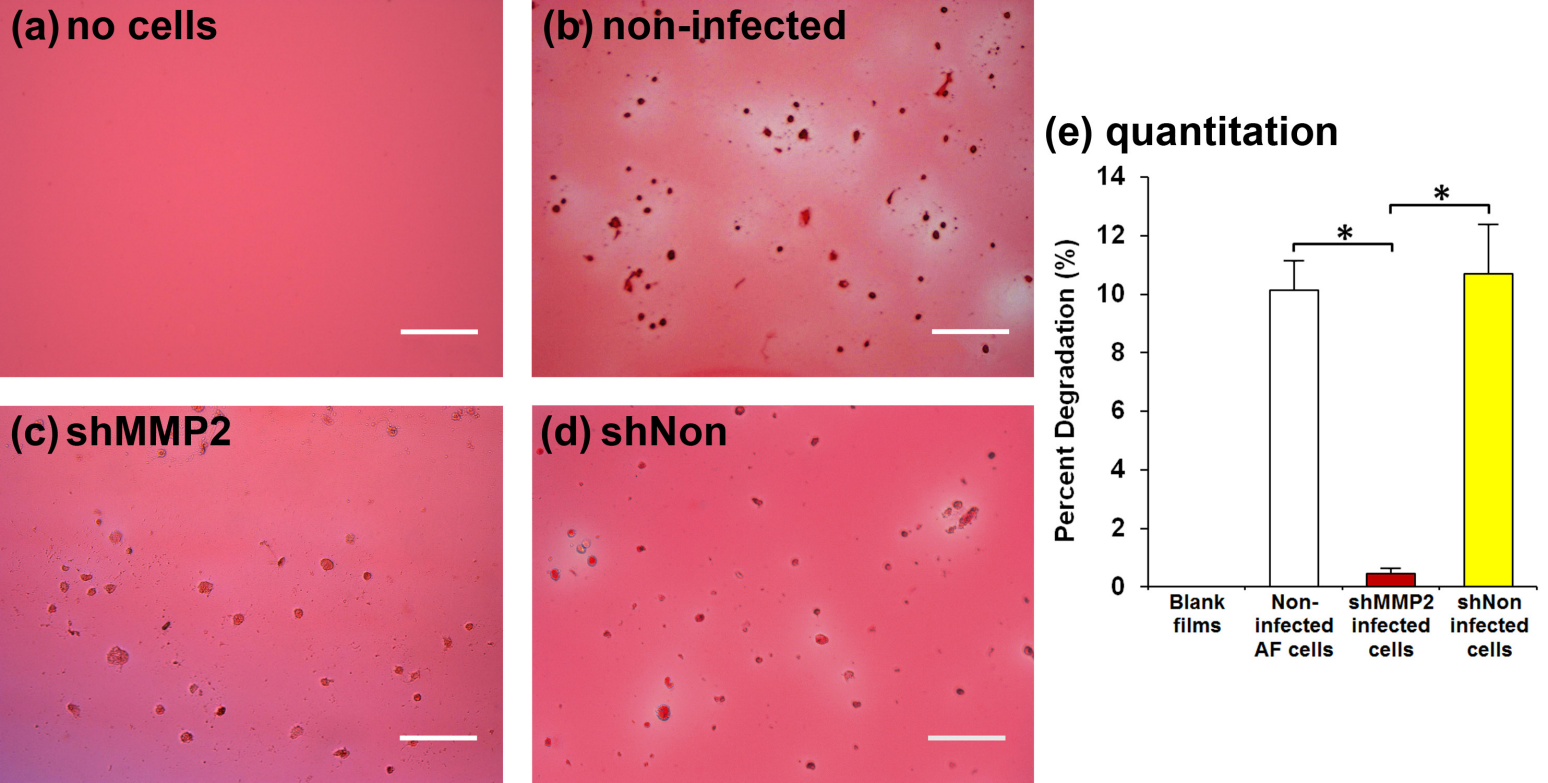
**shRNA construct against matrix metalloproteinase 2 (MMP-2) **(**shMMP2) inhibits localized gelatin degradation surrounding annulus fibrosus (AF) cells**. Microscopy images of gelatin films seeded with **(a) **no cells, **(b) **noninfected AF cells, **(c) **cells transduced with shMMP2, and **(d) **cells transduced with shNon. Films were stained at 4 days in culture with Ponceau S and visualized immediately. Scale bars represent 200 μm. All images were taken at 100×. Films with no cells (a) stained evenly red, whereas films seeded with noninfected AF cells (b) showed focal degradation of gelatin in the areas immediately surrounding cells, which is shown by the lighter-stained regions. Similar results are seen with cells transduced with shNon (d). Cells transduced with shMMP2 (c) show little to no focal degradation of gelatin, implying that MMP-2 is directly involved in gelatin breakdown. **(e) **Quantification of gelatin degradation. Gelatin films seeded with noninfected control AF cells and shNon-transduced cells were similar in percentage degradation of gelatin, which corroborated visual results. Compared with the shMMP2-transduced cells, degradation was significantly higher in the noninfected AF control group (*P *= 0.001) and shNon-transduced group (0.002), as observed visually.

To determine the functional effects of *MMP2 *RNAi, AF cells were plated on gelatin films to examine the extent and localization of degradative processes. After culture for 4 days, blank gelatin films (negative control) stained homogeneously red with Ponceau S (Figure 5a). In contrast, those seeded with noninfected control AF cells showed extensive degradation of the gelatin immediately surrounding the cells (Figure 5b). These areas appeared lighter pink to white, compared with the darker red-stained gelatin in areas farther away from cells. In films seeded with cells transduced with shMMP2, only very diffuse degrees of degradation were observed (Figure 5c). Films seeded with cells transduced with shNon exhibited patterns similar to those of noninfected controls, in which focal degradation occurred only immediately surrounding cells, although the degree of degradation was less (Figure 5d). Quantification of gelatin films verified visual results (Figure 5e), with noninfected cells and shNon-transduced cells having similar percentages of decrease in staining (10.1% and 10.7%, respectively), whereas shMMP2-transduced cells showed minimal change in staining (0.5%), significantly lower than both noninfected controls (*P *= 0.001) and shNon-transduced cells (*P *= 0.002). No clear differences were observed in the staining intensity of gelatin films away from cells, suggesting that MMP-2 is most effective in localized cell-mediated degradative processes.

### Collagen remodeling is impaired in AF cells deficient in MMP-2

**Figure 6:**
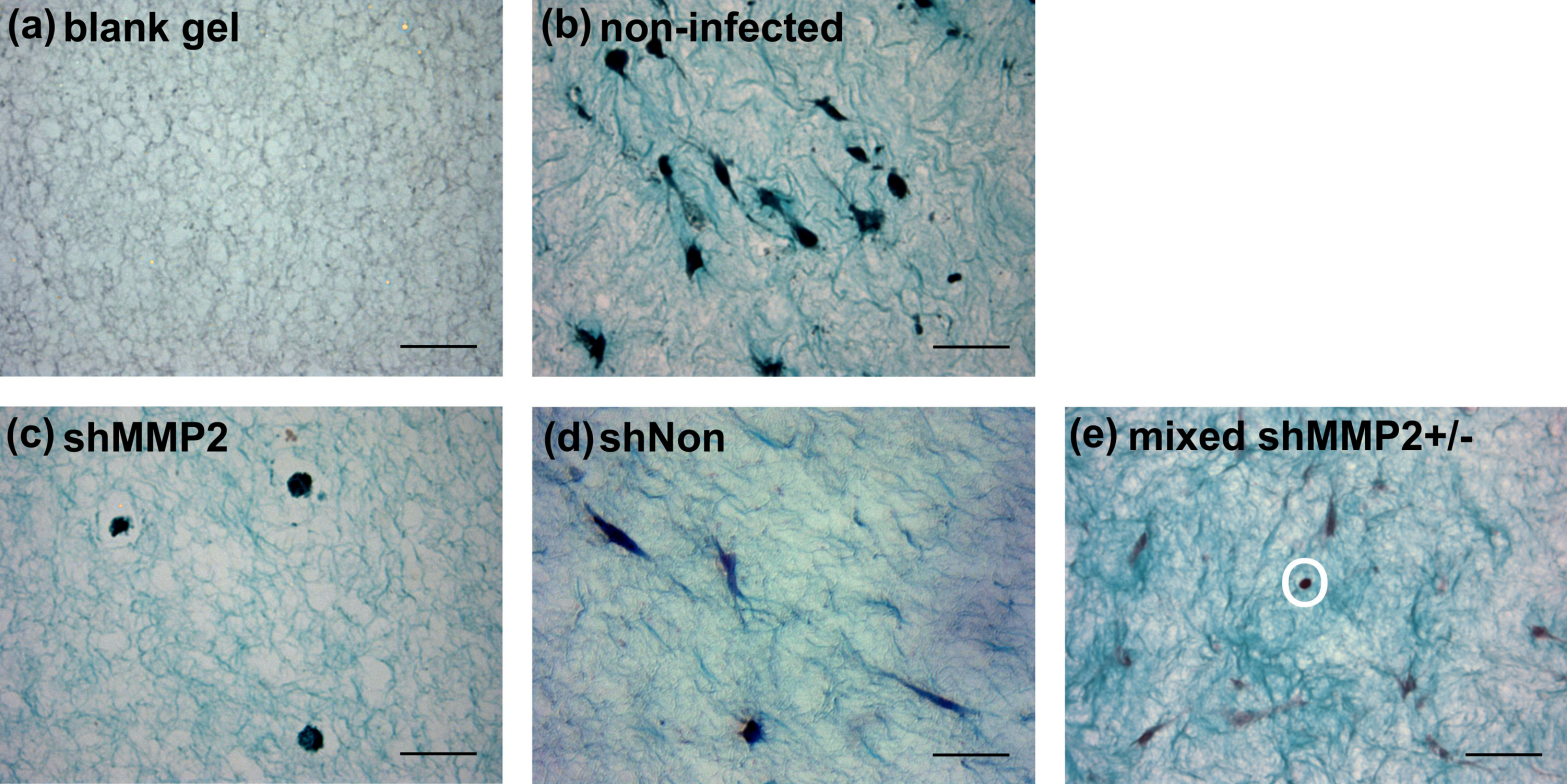
**shRNA construct against matrix metalloproteinase 2 (MMP-2) (shMMP2) impairs annulus fibrosus (AF) cell-mediated remodeling of collagen gels**. Histology images of collagen gels containing **(a) **no cells, **(b) **nontransduced AF cells, **(c) **a pure population of cells transduced with shMMP2, **(d) **a pure population of cells transduced with shNon, and **(e) **a mixed population of shMMP2-transduced and nontransduced cells. All gels were cultured for 7 days. Images for (a) through (d) were taken at 400×; scale bars represent 50 μm. Image for (e) was taken at 100×; scale bar represents 200 μm. Noninfected AF cells (b) and shNon-transduced cells (d) were shown to restructure collagen gels. These cells appeared elongated and anchored to the fibers. Cells transduced with shMMP2 (c) appeared similar to gels without cells (a), with little to no restructuring of the collagen network and rounded cells. In gels containing mixed populations of cells (e), collagen fibers were reorganized but rounded cells were clearly visible without reorganization of fibers in their immediate surroundings.

The ability for AF cells to reorganize and contract collagen gels was strongly diminished by *MMP2 *knockdown. Histologic staining of blank gels revealed a homogeneously porous structure throughout the gel (Figure 6a). In contrast, gels with noninfected control cells contained much denser regions of collagen surrounding cells, which were well integrated into the matrix. Collagen appeared to be organized into bundled structures throughout the entire gel (Figure 6b). In gels containing shMMP2-transduced cells, however, only minor differences in appearance were noted compared with blank gels (Figure 6c). Collagen remained free of fibrillar bundles, and was more homogeneously porous. Notably, cells were rounded and did not appear to interact with the collagen matrix. Gels containing shNon transduced cells also contained restructured collagen fibers with denser staining around the cells (Figure 6d). Those embedded with mixed populations of cells exhibited qualities similar to noninfected controls, but possessed areas containing rounded and unattached cells (Figure 6e).

Despite the striking histologic differences in collagen microstructure observed in the shMMP2 group compared with both noninfected and shNon, *MMP2 *silencing did not correspondingly affect collagen gel properties on the larger scale. In terms of macroscopic appearance, blank gel construct size remained the same size through 7 days of culture, whereas gels embedded with noninfected AF cells contracted approximately 83% relative to their original size. Gels embedded with cells transduced with shMMP2 contracted to a lesser degree (18%) than did gels with cells transduced with shNon (31%).

**Figure 7:**
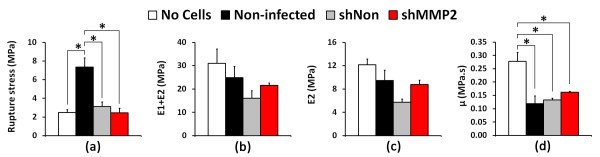
**shRNA construct against matrix metalloproteinase 2 (MMP-2) (shMMP2) inhibits strengthening of collagen gels by annulus fibrosus (AF) cells**. Parameters obtained from mechanical testing of collagen gels. **(a) **Rupture stress of collagen gels seeded with wild-type noninfected cells was significantly higher than that of acellular gels, gels populated with shNon-transduced cells, and gels populated with shMMP2-transduced cells (*P *< 0.05). **(b) **Short-term and **(c) **long-term moduli were lower in cell-seeded collagen gels but not significantly different from acellular gels. **(d) **Collagen gels seeded with cells possessed significantly lower viscous coefficients than did acellular gels (*P *< 0.05).

With respect to mechanical behavior, trends among groups were mixed. Noninfected wild-type AF cells significantly increased rupture stress of collagen gels (Figure 7a) compared with blank gels (*P *= 0.011), consistent with the notion that fibroblasts remodel and strengthen collagenous matrices. However, both shMMP2 and shNon control groups were unable to improve the rupture stress of collagen gels; values were comparable to acellular gels and were also significantly lower than those embedded with noninfected cells (*P *< 0.05). No clear trends in short-term (E_1 _+ E_2_) and long-term (E_2_) effective moduli of collagen gels were discernible, with differences among groups not statistically significant (Figure 7b, c). The act of seeding cells for all groups into collagen gels, however, resulted in a significant decrease (*P *< 0.05) in the viscous coefficient, μ (Figure 7d) for all groups. Noninfected AF cells and shNon transduced cells trended toward lower values compared with shMMP2 cells, but these differences were not statistically significant.

## Discussion

This study demonstrated that *MMP2 *expression is stimulated in the AF of IVDs subjected to overload or puncture injury in rodent caudal discs, and strongly supports the notion that MMP-2 plays a significant functional role in the degenerative changes previously observed in both animal models [16,19]. In particular, we showed that silencing *MMP2 *expression all but eliminated gelatinase activity in AF cells and profoundly compromised their ability to remodel collagen matrices. We also demonstrated that, similar to tumor invadopodia [20-23], MMP-2 preferentially functions in a localized region surrounding AF cells, rather than being secreted and acting at a distance. Contrary to our expectations, the impaired ability of AF cells to degrade and remodel their local microstructure did not translate to the macroscale characterization of the collagen-gel mechanical properties. The lack of clear trends indicates that bulk collagen-gel properties depend on more than just collagen microstructure. Others have similarly shown that collagen fiber alignment does not fully explain the direction-dependent material properties of collagen gels [24].

Various matrix-degrading enzymes have been found to be expressed at higher levels in degenerate human discs [2,9,25-29]. Although the biochemical aspects of MMP function have been elucidated, their specific functional roles in the multifaceted nature of the disorder are not well understood. For instance, it has been shown that MMPs can contribute not only to matrix degradation, but also to inflammatory conditions, innervation, and apoptosis [5,30,31] all of which have been observed in DDD. Although the precise coordination of the molecular events behind degenerative tissue remodeling remains unclear, demonstrating that MMP-2 is critical for localized matrix turnover by AF cells serves as an important step toward mechanistic understanding of DDD.

The observed effects in AF cells are consistent with the putative role of MMP-2 in other systems *in vivo*. Because gelatinases exhibit much greater enzymatic activity on denatured collagen than on native collagen [32], they are believed to work alongside other matrix-degrading enzymes in mediating radical changes in the ECM by removing bulk denatured collagen material (gelatin). Although MMP-2 is considered in most circles to function in the secondary processing of collagen, studies have shown its ability to cleave intact triple-helical collagen with some efficiency [5,33]. It is interesting to speculate whether MMP-2 might also serve a collagenolytic capacity in our collagen gel preparations, because the inability for shMMP2-transduced cells to remodel the gels was so dramatic. Further studies manipulating collagenase activity are needed to investigate the relative collagenolytic and gelatinolytic roles that potentially underlie MMP-2 function.

Changes mediated by MMP-2 can be either physiologic or pathophysiologic in context. For instance, MMP-2 and -9 have been implicated not only in development and repair, but also in pathologies of the tracheal gland [32], renal system [34,35], tumors [20,30,36], bone [37-39], and circulatory system [40,41], among others. In the current study, knocking down MMP-2 both impaired the localized degradation of gelatin and inhibited AF cells from remodeling the collagen matrix. This was evident in the histologic structure and contraction of collagen gels. Despite the known association between gelatinase activity and collagen gel remodeling, until now, no direct evidence suggested that these effects are directly coupled in AF cells.

This ambiguity stems in part from a complex relation that has been observed to exist among ECM-related cues, actin cytoskeletal organization, and MMP-2 activation. It has long been observed that cells can cause contraction of native collagen gels through reorganization of collagen fibrillar structure [42]. Generally, contraction of gels is accompanied by MMP-2 activation [43-47], which is thought to be linked with the suppression of stress fibers by collagen gel deformability [45,46,48]. Studies have also demonstrated that integrin binding is another critical factor in potentiating MMP-2 function [21,22,45,49-52].

Although these previous studies demonstrate an outside-in phenomenon in the regulation of MMP-2 activation by the collagen microenvironment, our data suggest that effects may be bidirectional. We found that pure populations of MMP-2-deficient AF cells remained rounded in collagen gels and were unable to remodel them, although cells remained adherent to gelatin films over several days. Thus, it appears that *MMP2 *expression may have profound influence over actin-cytoskeleton-dependent collagen gel remodeling, but no effect on cell-collagen binding, presumably via β1 integrins. Furthermore, it suggests that collagen gel contraction is more directly influenced by cytoskeletal organization than integrin ligation, which may be a necessary, but not sufficient, condition.

In terms of IVD physiology, our findings support the pursuit of further *in vivo *investigation. Evidence from the literature points to a potentially important role of MMP-2 in degradative changes in the AF. Across various species and insult techniques, animal models of degenerative changes in the IVD have demonstrated an association of MMP-2 with gross morphologic changes in the AF [3,10,53], which parallels observations in human discs during development and pathogenesis [2,27-29]. Consistent with these other studies, our data in mice and rats show that MMP-2 activity could increase relatively early in response to injurious load and annular puncture [16,19], suggesting possible involvement in the initial phases of degradation.

Experiments using *in vitro *cell-culture models provide some insight into the potential functional significance of MMP-2. Silencing *MMP2 *in primary AF cells abolished gelatinolytic activity in the vicinity of cells, implicating MMP-2 as the primary physiologic gelatinase responsible for localized breakdown of gelatin but of little relevance in distant matrix degradation. This observation is consistent with previous reports in the literature by others, that negligible MMP-9 is expressed in nonherniated human IVDs [28]. Collagen gel experiments underscore the functional significance of MMP-2 in structural reorganization of the ECM. Noninfected AF cells were able to modify the existing collagen scaffold into a more fibrous compact structure, resulting in gels that exhibited higher strength and lower viscosity, presumably due in part to the ability to remove denatured collagen. It also is possible that MMP-2 may contribute to the cells' ability to modify their biophysical environment through contraction, migration, and manipulation of collagen fibrils.

Several technical observations were also made during the course of this study. In our experiments, five shRNA sequences were tested for effectiveness in silencing *MMP2 *in rat AF cells. As expected, some constructs were more effective in silencing *MMP2 *than were others. It is known that siRNAs targeting different regions of the same gene vary markedly in their effectiveness [14], which depends on factors such as the secondary structure of the mRNA target, presence of the RNA-binding proteins, and thermodynamic stability of the duplex [14,54]. In mixed-populations cultures, it is also possible that the transduced and nontransduced populations exhibit differences in proliferation rates, although we did not measure this directly. It was, therefore, not unexpected that the level of knockdown might change over time.

Treating cells with blasticidin to obtain a pure population of only transduced cells eliminated any potential masking effects by nontransduced cells. Therefore, it was expected that the pure population would exhibit higher levels of knockdown compared with a mixed population. Instead, we observed less suppression of transcript levels in Experiments 2 and 3 for all of our constructs. It is possible that the 10-day blasticidin treatment used to kill nontransduced cells in these experiments may nonspecifically alter expression of other genes, as antibiotics in culture could interfere with multiple metabolic processes [55]. Despite the reduced silencing observed at the mRNA level, we continued to observe almost no protein expression.

Even though the shNon contained a scrambled sequence, some level of knockdown was observed in these cells as well. It is known that the interferon response may be triggered in the process of cell transfection with a viral vector [14,54] and may cause overall changes in the gene-expression profile in cells as well [56]. These factors together could lead to some level of gene knockdown observed in cells transduced with the shNon vector. In addition, some debate exists as to the specificity of sequences required for effective RNAi [57]. Various bases in the scrambled nonsense sequence may be able to match the *MMP2 *mRNA found in cells causing some knockdown effect. Compared with the nonsense construct, however, shMMP2 was significantly more effective in reducing mRNA and protein levels. Therefore, the chosen sequence for shMMP2 was considered effective for use in future RNAi experiments.

Although more work is needed, elucidating the functional roles of degradative enzymes in degenerative remodeling of the disc ECM contributes to the identification of potential targets for gene therapy. RNAi technology offers the advantage of stable silencing of catabolic factors, which can be of therapeutic value in treating disc degeneration. To this extent, lentiviral vectors have provided an advancement in RNAi and offer the means to achieve significant levels of gene transfer both *in vitro *and *in vivo *[54]. Various studies have used siRNAs for RNAi in IVD cells [58,59], but to our knowledge, this study is the first to use shRNAs to ascertain the functional role of a molecule putatively involved in disc degeneration. *MMP2 *as a gene-therapy target has already been studied *in vivo *by using animal models as a strategy to treat tumors. In nude mice, siRNAs against *MMP2 *resulted in decreased tumor invasion, migration, and angiogenesis, with a 60% reduction in tumor size [60,61]. This approach allows only transient downregulation of gene expression, making it difficult to adapt for therapeutic purposes. However, shRNA delivery can allow long-term studies to determine the functional role of genes relevant to intervertebral disc degeneration, such as *MMP2*.

## Conclusions

Taken together, results from this study suggest that the increased presence of MMP-2 in animal models of disc degeneration may be associated with the degradative changes that are observed in the AF. Whether a similar relation might exist in degradative changes in human DDD remains to be determined. By using RNAi, we showed that MMP-2 functions as the primary gelatinase and is used by AF cells for degrading the local surrounding matrix. Notably, in the absence of MMP-2, collagen remodeling is significantly impaired, suggesting that it may play a significant role in collagen turnover and structural alterations in the AF. This mechanism could offer one avenue for therapeutic intervention against degenerative disc disease.

## Abbreviations

AF: annulus fibrosus; DDD: degenerative disc disease; DMEM: Dulbecco Modified Eagle Medium; ECM: extracellular matrix; FBS: fetal bovine serum; IVD: intervertebral disc; MMP: matrix metalloproteinase; PBS: phosphate-buffered saline; RNAi: RNA interference; RT-PCR: reverse transcriptase polymerase chain reaction; shRNA: small-hairpin RNA.

## Competing interests

The authors declare that they have no competing interests.

## Authors' contributions

AR performed all molecular cloning, cell experiments, mechanical testing, and histology. HK performed animal surgeries and subsequent IHC and microscopy. JDT contributed to gene expression and collagen gel-testing data analysis. AR, HK, JDT, and AHH interpreted the data. AHH and HK provided statistical analyses. AR and AHH conceived and designed all aspects of this study and wrote the manuscript with support from JDT and HK. All authors read, revised, and approved the final manuscript.
